# Redefining the draft pick valuation in the National Football League

**DOI:** 10.3389/fspor.2025.1628223

**Published:** 2025-09-10

**Authors:** Bryce Hadley, Jun Woo Kim, Marshall Magnusen, Kyoung Tae Kim

**Affiliations:** ^1^ School of Sport, Tourism and Hospitality Management, Temple University, Philadelphia, PA, United States; ^2^ School of Global Business, Arcadia University, Glenside, PA, United States; ^3^ Department of Educational Leadership, Baylor University, Waco, TX, United States; ^4^ Department of Health and Human Performance, Kean University, Union, NJ, United States

**Keywords:** NFL, draft pick, pick value chart, chart, trade up

## Abstract

The National Football League (NFL) Draft plays a critical role in determining team compositions and enhancing the competitive balance within the league. This study examines the valuation dynamics of NFL Draft picks, contrasting traditional valuation paradigms like Jimmy Johnson's Pick Value Chart (PVC) to estimate the intrinsic value of draft selections. Utilizing metrics such as weighted approximate value (wAV), games played (GP), and seasons started (ST), we derived that late-round draft picks might be undervalued in the conventional PVC. Our regression results indicated that the final pick in the first round holds a value closer to 56% of the first pick's worth, contrasting sharply with PVC's 20%. Our refined model suggested that the value of a pick 200 is about 30 times more than what PVC projects. We incorporate round-specific “Traded Up” dummy variables into our player-level regression models to test whether trade-up selections yield better outcomes. Results reveal that early round trade-ups, particularly in Round 1, are associated with significantly lower player performance, suggesting inefficiencies driven by overconfidence. In contrast, trade-ups in Round 4 show a modest positive effect, while other rounds yield no consistent advantage. These findings indicate that teams may benefit more from accumulating late-round picks than from aggressively trading up early. This paper accentuates the need for NFL teams to integrate data-driven models for informed draft decisions, thereby deepening insights into NFL draft valuation.

## 1 Introduction

Since its inception in 1920, Sundays in fall have become synonymous with the National Football League (NFL), a cultural juggernaut that generated over $18 billion in revenue during the 2021 season (1) and accounts for 82 of the top 100 most-watched telecasts in the United States (2). Each offseason, the NFL Draft—now a seven-round event in which each of the 32 teams receives one pick per round—determines how collegiate talent is allocated, with each selection representing four guaranteed contract years (plus a fifth-year option for first-rounders) under the Collective Bargaining Agreement (3, 4). The draft order itself is structured to promote competitive balance: the previous season's worst team picks first, while the Super Bowl champion picks last in each round.

Instituted in 1936 to level the playing field among franchises (5), the draft remains central to roster-building strategies. Some contenders—like the 2021 Super Bowl-winning Los Angeles Rams—have traded high draft capital for proven veterans (e.g., Matthew Stafford, Von Miller, Jalen Ramsey), while others—such as the Cincinnati Bengals—have doubled down on early picks to land transformative players (Joe Burrow, Ja'Marr Chase, Jessie Bates). The divergent post-season trajectories of those teams—Rams missing the playoffs the following year versus Bengals returning to the AFC Championship—underscore the enduring question of how teams should value and leverage draft assets.

Jimmy Johnson's Pick Value Chart (PVC) has long served as the NFL's heuristic for trading draft capital, assigning 3,000 points to the first overall pick and descending to just 2 points by pick 224 (6). Though most franchises maintain their own proprietary overlays [often rescaling the top pick to 1,000 rather than 3,000; (7)], retrospective analyses—such as the Minnesota Vikings' 2023 trade for Pick 141 (35.5 points) in exchange for Picks 158 and 211 (35.2 points combined)—reveal that draft-day swaps generally adhere closely to PVC valuations, even as questions linger about its middle-round accuracy.

A growing body of work calls for data-driven refinements. Massey and Thaler (8) showed that Johnson's chart overvalues first-rounders relative to later selections, while Duquette and Cebula (3) used weighted career Approximate Value (AV) and polynomial regression to demonstrate that PVC underestimates post-first-round worth—finding, for instance, a mere 67-point AV drop from Pick 1–2 vs. PVC's 400-point gap. Schuckers (6) further compared games played, games started, AV, and Pro Bowl appearances (1991–2001), applying logarithmic models to assign greater value to late-round picks than earlier AV-based charts. Together, these studies highlight AV's strength as a continuous, position-neutral metric and underscore the need to recalibrate Day 3 values (i.e., Rounds 4–7).

One notable inadequacy that has been overlooked in previous studies is the absence of consideration for the history of draft trades. Instead, the focus has predominantly been on assessing the individual player's AV at a specific draft pick. Hersch and Pelkowski (9) introduced a “Traded Up” binary indicator—1 if a team packaged assets to move up, 0 otherwise—to capture the observed premium of trade-up selections during their first three NFL seasons. In this study, we employ a more refined empirical strategy by incorporating round-specific “Traded Up” dummy variables into our player-level regression models. This allows us to assess whether trading up in the first round (where players are expensive and carry high expectations) differs from trading up in later rounds (where teams may be seeking undervalued depth).

To capture player value comprehensively, we analyze three performance metrics: weighted Approximate Value (wAV), games played, and seasons started. These outcomes offer a multidimensional perspective, reflecting the complexity of long-term player contributions. The central research question guiding this study is: “What is the value of an NFL Draft pick relative to others, and how do trade-ups influence this valuation?” Our study builds on prior work by updating the data, introducing a round-specific treatment of trade-ups, and expanding outcome measures. We aim to provide a more detailed and data-driven framework to help NFL teams assess draft pick value and inform roster construction strategy.

## 2 Methods

### 2.1 Data collection

The data were retrieved from publicly available information provided by pro-football-reference.com. The complete dataset was comprised of (a) draft and trade information (year, team, draft pick, and trade up), (b) game information (the number of games played, and the number of seasons played as a starter), and (c) player performance statistics throughout their career (wAV, yds, tackles, etc.). The trade-up variable is defined as a set of seven binary indicators—TradeUp_Rnd1 through TradeUp_Rnd7—each representing whether a team traded up to select a player in a specific round of the draft. For the draft pick variable, a lower numerical value indicates an earlier selection in the draft process. As suggested by Hersch and Pelkowski (9) and Duquette and Cebula (3), the draft pick is viewed as a primary determinant of player contribution, regardless of which team makes the selection. Their research also posits the inclusion of a quadratic term to account for the talent pool disparities. For instance, in a 32-team draft, the anticipated performance differential between the first and second draft picks might be more pronounced than that between the 31st and 32nd picks. To encapsulate this curvilinear relationship, a polynomial regression can be adopted. We initiated with a lower-order polynomial regression and progressed as suggested by Duquette and Cebula. A fifth order polynomial, which yielded the best goodness-of-fit, was employed to estimate the impact of draft selection on player career achievements.

The metric of wAV (weighted career approximate value) refines the AV metric by emphasizing a player's best seasons rather than aggregating a player's AV for each season of their career. Thus, wAV is more generous to players who shine exceptionally in certain seasons than those who have lengthier but less remarkable careers. We favored the wAV mainly to equitably juxtapose players from varied positions. The distinct roles of, say, a quarterback and a linebacker make conventional statistics inadequate for direct comparison. The wAV addresses this challenge by presenting a position-neutral assessment tool. Notably, Pro-Football-Reference calculates wAV by distributing a team's total AV among its starters, based on positional weights and performance, thereby producing a robust indicator of relative value. This allows for fairer cross-positional comparisons and aligns well with our model's objective of capturing player impact beyond raw counting stats.

The total number of observations for the final dataset included 4,996 distinct draft picks spanning from 1993 to 2012. This time frame was selected based on the consistency in the number of draft picks across these years. Furthermore, as 2012 is more than a decade ago, the majority of players from that year have since retired, solidifying their wAV statistics. Observations lacking data in weighted approximate value, games played, or seasons started were excluded.

Upon finalizing the primary dataset, it became imperative to compute the averages for each draft pick to properly run the models. This involved calculating the mean wAV for each 1st overall pick, then methodically doing the same for every subsequent pick in the NFL Draft. That is, we calculated the average wAV for all 262 slots over the 20 years of NFL Draft from 1994 to 2012. We employed the approach of Duquette and Cebula, who calculated the average AV for individual draft pick selections spanning from 1994 to 2003.

### 2.2 Empirical model 1

The first model used in this study aimed to estimate the average wAV of a draft pick based on their specific draft selection. In the first model, we adopted a framework of the draft order value developed by Duquette and Cebula (3) to offer a new pick value chart by incorporating a new set of performance value calculations. Duquette and Cebula used a polynomial regression model to create their pick value chart, and the results showed that the fifth-order polynomial regression yielded a satisfying goodness-of-fit. Accordingly, we adopted the fifth-order polynomial metric to estimate the best predictive regression lines for our proposed models. To analyze the influence of pick selection on wAV, we implemented a polynomial regression model. This model was selected to elucidate the nonlinear relationship between the draft pick number and wAV. It is important to note that the number of data-points was compressed to 262 by averaging the values for all 262 NFL Draft pick slots over the 20-year study period under consideration. The estimating Equation 1 is presented as follows:

(1) $$\begin{array}{rl} \mathrm{wAV} & =B0+B1\times \mathrm{Pick}+B2\times \mathrm{Pic}k^{2}+B3\times \mathrm{Pic}k^{3}+B4\\ & \times \mathrm{Pic}k^{4}+B5\times \mathrm{Pic}k^{5}+e\; \end{array}$$

where the dependent variable, wAV is explained by the pick number and its exponentials multiplied by the coefficient *Bi*.

Drawing upon prior research, there are additional factors to consider when determining the value of a draft pick. By incorporating metrics such as a number of games played (GP) and a number of seasons played as a starter (ST), we aim to refine and replicate the existing model to forecast both the average number of games played and seasons initiated. Schuckers' study (6) notably highlighted the significance of the “games played” and “seasons started” variables. The corresponding models are specified in Equations 2, 3:

(2) $$\begin{array}{rl} \mathrm{GP} & =B0+B1\times \mathrm{Pick}+B2\times \mathrm{Pic}k^{2}+B3\times \mathrm{Pic}k^{3}+B4\\ & \times \mathrm{Pic}k^{4}+B5\times \mathrm{Pic}k^{5}+e \end{array}$$

(3) $$\begin{array}{rl} \mathrm{ST} & =B0+B1\times \mathrm{Pick}+B2\times \mathrm{Pic}k^{2}+B3\times \mathrm{Pic}k^{3}+B4\\ & \times \mathrm{Pic}k^{4}+B5\times \mathrm{Pic}k^{5}+e \end{array}$$

where the dependent variables, GP or ST are explained by the pick number and its exponentials.

### 2.3 Empirical model 2

The second model in this study examines the implications of trading up in the NFL Draft and its impact on player value across different draft pick rounds. Building upon the approach introduced by Hersch and Pelkowski (9), we explored whether the effect of a trade-up decision is uniform across all rounds or whether its impact varies depending on when in the draft it occurs. They explained that players obtained through trade-up mechanisms tend to offer a more substantial contribution to their teams compared to those selected in regular draft positions. We extended this by introducing a set of interaction terms to capture the round-specific effects of trading up. To achieve this, we constructed a regression model that encompasses all player data (*n* = 4,996), deviating from merely averaging individual pick numbers. Specifically, we generated seven dummy variables (TradeUp_Rnd1 through TradeUp_Rnd7), each indicating whether a trade-up occurred in the corresponding draft round. This structure allows us to differentiate the marginal effect of a trade-up decision by round and to evaluate whether early-round trade-ups, for example, carry different implications than those in later rounds. The corresponding models are specified in Equations 4–6:

(4) $$\begin{array}{rl} \mathrm{wAV}= & \;B0+B1\times \mathrm{Pick}+B2\times \mathrm{Pic}k^{2}\\ + & \;B3\times \mathrm{Pic}k^{3}+B4\times \mathrm{Pic}k^{4}+B5\times \mathrm{Pic}k^{5}\\ + & \overset{7}{\underset{r=1}{\sum }}\gamma r\times \mathrm{TradedUp}\_\mathrm{Round}r+e \end{array}$$

(5) $$\begin{array}{rl} \mathrm{GP}= & \;B0+B1\times \mathrm{Pick}+B2\times \mathrm{Pic}k^{2}+B3\times \mathrm{Pic}k^{3}+B4\times \mathrm{Pic}k^{4}\\ & +B5\times \mathrm{Pic}k^{5}+\overset{7}{\underset{r=1}{\sum }}\gamma r\times \mathrm{TradedUp}\_\mathrm{Round}r+e \end{array}$$

(6) $$\begin{array}{rl} \mathrm{ST}= & \;B0+B1\times \mathrm{Pick}+B2\times \mathrm{Pic}k^{2}+B3\times \mathrm{Pic}k^{3}\\ + & \;B4\times \mathrm{Pic}k^{4}+B5\times \mathrm{Pic}k^{5}\\ + & \overset{7}{\underset{r=1}{\sum }}\gamma r\times \mathrm{TradedUp}\_\mathrm{Round}r+e \end{array}$$

where the dependent variables, wAV, GP, and ST, are functions of the pick number, its exponentials, and the “Traded Up” variable.

In summary, our second models dive into the repercussions of trading up within the NFL Draft on the perceived value of a draft pick, drawing inspiration from Hersch and Pelkowski's (9) findings. Their research suggested superior performance and contribution from players acquired via trade-up strategies, as opposed to those chosen in standard draft sequences. By incorporating round-specific trade-up interactions, we aim to offer a more precise understanding of when such strategic moves add value—and when they may not.

## 3 Results

The data reveal that, on average, a draft pick commences as a starter for approximately 2.3 years in the NFL. The average wAV for a player's career is around 18. Furthermore, NFL Draft selections typically partake in an average of 65 games over the span of their professional careers.

### 3.1 Draft pick value model

**Figure 1:**
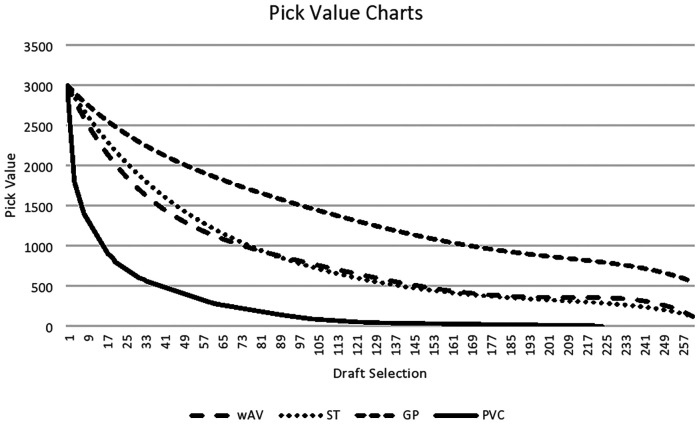
Comparison of PVC with new metrics from Model 1. 1993–2012 drafted players included. WAV, player’s weighted approximate value; GP, games played; St, season started.

In our Model 1, the three key metrics predicted by a player's draft position serve as insightful indicators for gauging a player's value over their career. As Schuckers (6) revealed previously, none of our metrics in Model 1 align with the PVC valuation approach introduced by Jimmy Johnson. That is, each metric has its unique merits. Specifically, games played (GP) variable values players based on their contributions to the team. Seasons started (ST) metric represents the number of seasons during which a player was part of the primary lineup, indicating his consistent value to the team over time. The weighted approximate value (wAV) allocates a proportionate value to a player based on the team's yearly performance and computes a weighted aggregate over his career. As illustrated in Figure 1, when compared to PVC, each metric is assessed against the fifth-order polynomial matrix for draft position. To refine the data representation, we averaged data-points across the 262 NFL draft pick slots spanning the 20-year study period, effectively compressing the dataset to 262 points. Remarkably, our fifth-order polynomial regression achieved a R^2^ of 0.90 for weighted approximate value (wAV), 0.82 for games played (GP), and 0.90 for season started (ST) (see Table 1). Model 1 predicts that a player selected 10th overall would have a career wAV of approximately 51.5, while a player selected 100th would have a predicted wAV closer to 16.4—highlighting the steep early-round dropoff in expected value.

**Table 1:** Regression results of Model 1.

Variable	Model 1 (DV = wAV)			Model 1 (DV = GP)			Model 1 (DV = ST)		
	*β*	S.E.	*P*-value	*β*	S.E.	*P*-value	*β*	S.E.	*P*-value
Draft	−1.352	0.130	0.001	−1.436	0.382	0.001	−0.131	0.018	0.001
Draft^2	0.018	0.003	0.001	0.015	0.009	0.099	0.001	0.001	0.005
Draft^3	−0.001	0.001	0.001	−0.001	0.001	0.201	−0.001	0.001	0.104
Draft^4	0.001	0.001	0.001	0.001	0.001	0.241	0.001	0.001	0.221
Draft^5	−6.63 × 10^−10^	0.001	0.001	−0.001	0.001	0.256	−0.001	0.001	0.287
Constant	63.432	1.698		136.445	5.010		8.115	0.235	
*R* ^2^	0.902			0.821			0.903		

The dataset was compressed to 262 observations by averaging values for each of the 262 NFL Draft pick slots over the 20-year period.

DV, dependent variable; SE, standard errors; wAV, player's weighted approximate value; GP, games played; ST, seasons started.

We have recalibrated the PVC using our new metric, wAV. Originally conceived in the 1990s, the traditional PVC outlines the relative worth of every NFL draft pick for trading intentions, anchoring the first overall pick at a value of 3,000. To derive a modern PVC using wAV, we employed a multiplier ensuring that the premier overall pick retained a value of 3,000, consistent with Jimmy Johnson's PVC standard. This multiplier was determined by dividing the regression value of the first pick by 3,000. Table 2 furnishes our recommended PVC for all 262 draft picks. This revised PVC facilitates direct comparisons with other established charts, including Jimmy Johnson's PVC and Duquette and Cebula's PCV. Notably, our pick values exhibit a less pronounced decline compared to Jimmy Johnson's original model, suggesting that the latter have undervalued lower draft selections.

**Table 2:** Proposed pick value chart.

Round 1		Round 2		Round 3		Round 4		Round 5		Round 6		Round 7	
Pick	Value	Pick	Value	Pick	Value	Pick	Value	Pick	Value	Pick	Value	Pick	Value
1	3,000	33	1,647	65	1,095	97	818	129	605	161	439	193	364
2	2,937	34	1,621	66	1,084	98	811	130	599	162	436	194	363
3	2,876	35	1,596	67	1,074	99	803	131	593	163	432	195	362
4	2,817	36	1,572	68	1,063	100	796	132	587	164	428	196	361
5	2,759	37	1,549	69	1,053	101	789	133	581	165	424	197	361
6	2,702	38	1,526	70	1,042	102	782	134	575	166	421	198	360
7	2,647	39	1,504	71	1,032	103	775	135	569	167	417	199	360
8	2,594	40	1,482	72	1,023	104	768	136	564	168	414	200	360
9	2,542	41	1,461	73	1,013	105	761	137	558	169	411	201	359
10	2,491	42	1,441	74	1,004	106	755	138	552	170	408	202	359
11	2,442	43	1,421	75	994	107	748	139	546	171	405	203	359
12	2,394	44	1,402	76	985	108	741	140	541	172	402	204	358
13	2,348	45	1,383	77	976	109	734	141	535	173	399	205	358
14	2,303	46	1,365	78	967	110	727	142	530	174	396	206	358
15	2,259	47	1,347	79	958	111	721	143	524	175	394	207	358
16	2,216	48	1,330	80	950	112	714	144	519	176	391	208	358
17	2,174	49	1,313	81	941	113	707	145	514	177	389	209	358
18	2,134	50	1,297	82	933	114	701	146	509	178	386	210	358
19	2,094	51	1,281	83	925	115	694	147	503	179	384	211	358
20	2,056	52	1,265	84	917	116	688	148	498	180	382	212	358
21	2,019	53	1,250	85	909	117	681	149	493	181	380	213	358
22	1,983	54	1,235	86	901	118	675	150	488	182	378	214	358
23	1,948	55	1,221	87	893	119	668	151	483	183	376	215	357
24	1,914	56	1,207	88	885	120	662	152	479	184	375	216	357
25	1,880	57	1,193	89	877	121	655	153	474	185	373	217	357
26	1,848	58	1,180	90	870	122	649	154	469	186	372	218	357
27	1,817	59	1,167	91	862	123	643	155	465	187	370	219	357
28	1,786	60	1,155	92	854	124	636	156	460	188	369	220	356
29	1,757	61	1,142	93	847	125	630	157	456	189	368	221	356
30	1,728	62	1,130	94	840	126	624	158	452	190	367	222	355
31	1,700	63	1,118	95	832	127	618	159	448	191	366	223	355
32	1,673	64	1,107	96	825	128	611	160	443	192	365	224	354

Among the three regression models, the season started (ST) metric exhibits the most subtle downward trend in pick value. Interestingly, the higher the exponent applied to the pick number, the less significant the resulting variable appears to be. Comparing our value chart in Figure 1 to Jimmy Johnson's Pick Value Chart, it's evident that our trajectory is more gradual. For instance, while Jimmy Johnson's chart dictates that the final pick of the first round holds 20% of the value of the initial pick, our games played (GP) metric suggests a value near 56%. Duquette and Cebula's (3) analysis places this value at approximately 50%. By the culmination of the second round, Jimmy Johnson's PVC posits that the round's concluding pick holds a value of 9%. In contrast, our analysis ascribes it a value of 37%, closely mirroring Duquette and Cebula's estimation of 36%. Among our metrics, the weighted approximate value (wAV) and season started (ST) metrics display remarkable similarities, positioning themselves centrally when compared to the other models. A consistent pattern in these models is their inclination to value late-round picks more than Jimmy Johnson's model does. Notably, our pick values consistently outpace the original PVC for every draft pick across all three metrics. Figure 1 offers a side-by-side comparison of our metrics against Jimmy Johnson's PVC (see Figure 1).

### 3.2 Draft pick value model with traded up variable

Upon examining the outcomes from Model 2 using round-specific trade-up indicators, the model reveals meaningful variation in the impact of trade-up decisions depending on the round in which the selection occurred. The R-squared values for Model 2 stand at approximately 0.31 for weighted approximate value (wAV), 0.22 for games played (GP), and 0.31 for season started (ST). While these figures are markedly lower than the consistent values within the 0.8–0.9 range observed in Model 1, they are consistent with expectations from models using disaggregated player level data. The regression results show that trading up in the first round is consistently associated with significantly lower performance outcomes across all three metrics (see Table 3). Specifically, round 1 trade-ups correspond with lower wAV scores, fewer games played, and fewer seasons started—challenging the notion, suggested by Hersch and Pelkowski (9), that trading up universally enhances player value. These findings indicate that early-round trade-ups may often be overpriced or inefficient, potentially driven by overconfidence in the expected performance of high-profile prospects. Interestingly, trade-ups in round 4 yielded a statistically significant positive effect on seasons started, suggesting that certain mid-round trades may yield undervalued talent or better returns on investment. For all other rounds, the trade-up indicators were statistically insignificant.

**Table 3:** Regression results of model 2.

Variable	Model 2 (DV = wAV)			Model 2 (DV = GP)			Model 2 (DV = ST)		
	*β*	S.E.	*P*-value	*β*	S.E.	*P*-value	*β*	S.E.	*P*-value
Draft	−1.316	0.139	0.001	−1.193	0.3601	0.001	−0.122	0.019	0.001
Draft^2	0.016	0.003	0.001	0.007	0.008	0.425	0.001	0.001	0.081
Draft^3	−0.001	0.001	0.001	−0.001	0.001	0.785	−0.001	0.001	0.595
Draft^4	0.001	0.001	0.001	0.001	0.001	0.939	0.001	0.001	0.937
Draft^5	−0.001	0.001	0.002	0.001	0.001	0.974	0.001	0.001	0.896
TradeUp_Rnd1	−5.442	2.186	0.013	−13.328	5.673	0.018	−0.815	0.310	0.008
TradeUp_Rnd2	−0.848	2.186	0.698	−5.309	5.671	0.349	−0.065	0.309	0.833
TradeUp_Rnd3	−0.364	2.798	0.896	−3.045	7.259	0.674	0.074	0.396	0.851
TradeUp_Rnd4	3.646	2.534	0.150	10.229	6.574	0.119	0.919	0.359	0.011
TradeUp_Rnd5	1.677	2.574	0.514	9.609	6.677	0.150	0.385	0.365	0.291
TradeUp_Rnd6	1.419	4.385	0.746	4.196	11.377	0.712	0.845	0.621	0.173
TradeUp_Rnd7	−1.371	5.186	0.791	−3.646	13.453	0.786	−0.277	0.735	0.705
Constant	63.993	1.796		136.567	4.658		8.170	0.254	
*R* ^2^	0.310			0.223			0.307		

DV, dependent variable; SE, standard errors; wAV, player's weighted approximate value; GP, games played; ST, seasons started.

**Figure 2:**
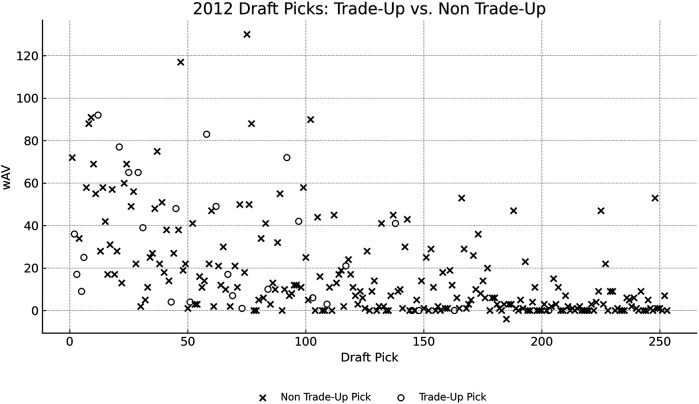
Trade-Up vs. Non-Trade-Up picks in 2012. wAV, player's weighted approximate value.

Figure 2 visualizes 2012 draftees' wAV by draft position, distinguishing players selected via trade-up from those who were not. The visual inspection reveals no clear systematic advantage in wAV for players acquired through trade-up strategies. In fact, some mid-to-late round players not acquired via trade-up achieved comparable or higher wAV outcomes than their trade-up counterparts. This dispersion supports our regression findings: the “Traded Up” variable generally lacks statistical significance across most rounds. The only exception was a modest positive effect observed in Round 4. the patterns observed in 2012 are consistent with those found in other draft years within our dataset, reinforcing the broader conclusion that trade-up decisions do not reliably yield superior performance outcomes.

Taken together, these results reaffirm prior findings from Duquette and Cebula (3) and Schuckers (6), while also highlighting the importance of disaggregating trade-up effects by round. NFL teams appear to systematically overvalue early-round trade-ups and undervalue later selections. These insights suggest a potential strategic opportunity to extract more value from the draft by prioritizing later-round selections—particularly through trade-down strategies—rather than aggressively pursuing early-round trade-ups.

## 4 Discussion

From our findings, we conclude the PVC that was created by Jimmy Johnson has indeed underestimated the value of late round draft picks relative to their early NFL draft picks. Consistent with Duquette and Cebula (3), we find strong evidence that Rounds 4–7 produce players who outperform their conventional point-based draft valuation. In particular, our updated chart shows that players selected with later picks—such as Pick 200—consistently generate greater career value than the PVC suggests. Our findings also highlight that wAV and seasons started were the two measures that had the highest variance predicted by the proposed models, thereby suggesting that they provide more precise indicators of player success.

The introduction of round-specific “Traded Up” dummy variables provides a more refined lens to assess the impact of draft-day trades. Although these variables were not consistently statistically significant across all rounds, their inclusion revealed nuanced trends. Notably, early-round trade-ups were often associated with diminished player outcomes, while certain mid-round trade-ups (e.g., Round 4) showed modest positive effects. These results challenge the prevailing assumption that trading up inherently improves draft efficiency, and instead highlight the potential value of accumulating later-round picks.

While our focus was on evaluating the impact of trading up, it's important to note that teams trading down may also benefit strategically. By acquiring more picks, particularly on Day 3, they enhance their chances of securing undervalued talent and managing roster costs effectively under a hard salary cap. This tradeoff can be especially beneficial in deep drafts or for franchises prioritizing long-term depth over short-term impact. The noteworthy difference exists between the PVC created in this study and Johnson's model, particularly illustrated by the variation between the first round and all subsequent rounds, a characteristic that is markedly more dramatic in Johnson's Model. The results displayed in the charts from this study shows a high degree of similarity to those from Duquette and Cebula's (3) model. In the models derived from this study, the values appear closer in the first round, yet exhibit approximate the same difference in the later rounds.

By leveraging metrics such as the weighted approximate value (wAV), games played (GP), and season started (ST), we present a more nuanced approach to understanding player value over their careers. Our findings highlight the potential undervaluing of lower draft selections in conventional models like that of Jimmy Johnson. The insights derived here not only offer a recalibrated PCV but also emphasize the evolving landscape of player assessment in the NFL. As the dynamics of sport shift and strategies become more sophisticated, it becomes imperative for teams to rely on data-driven, updated models to make informed decisions. This approach ensures that their draft choices yield the best possible outcomes and maximize the return on their draft investments.

The practical implications of this study are expected to be reflected in the NFL Draft boards across the league. In light of this study's findings, teams might consider concentrating more heavily on Day 3 selections (Rounds 4–7) to find the true value picks for their franchises. That is, Day 3 picks are undervalued by the existing draft pick value charts, and teams can accumulate many late draft picks by trading down during the draft. The influence of such studies on the strategic considerations of NFL teams is evidenced by the trading behaviors of teams like the 49ers and Rams, who traded their top draft picks for proven players such as Christian McCaffery and Von Miller. Both teams have also stockpiled numerous Day 3 picks that they have used to reload their team with players. This strategy has helped the Rams win Super Bowl LVI, and also facilitated the 49ers' appearances in three of the past four NFC Championship games. This strategy aligns well with the recommendations derived from this study.

While our findings underscore the strategic merit of targeting Day 3 selections from a performance-value standpoint, we also recognize that actual draft behavior may be shaped as much by financial considerations as by performance projections. Teams like the 49ers and Rams, for instance, may pursue more Day 3 picks not solely because they are analytically undervalued, but because they offer cost-controlled talent essential for balancing rosters filled with high-priced veterans acquired through free agency or trade.

In conclusion, this research contributes valuable insights into the intricate dynamics of the NFL draft selection process. We highlight the potential underestimation of late-round picks' value and highlights the effectiveness of certain strategic choices, as evidenced by the success of teams like the Rams and 49ers. These findings should stimulate further contemplation and strategic evolution among NFL teams, enhancing their decision-making processes and potentially altering long-standing norms in draft pick selections. As the field of sports analytics continues to advance, studies in draft pick valuation will remain instrumental in informing and refining these crucial aspects of a team's roster management.

## Data Availability

Publicly available datasets were analyzed in this study. This data can be found here: pro-football-reference.com.
